# Physical Activity, Energy Expenditure, and Defense of Body Weight in Melanocortin 4 Receptor-Deficient Male Rats

**DOI:** 10.1038/srep37435

**Published:** 2016-11-25

**Authors:** Tariq I. Almundarij, Mark E. Smyers, Addison Spriggs, Lydia A. Heemstra, Lisa Beltz, Eric Dyne, Caitlyn Ridenour, Colleen M. Novak

**Affiliations:** 1 College of Agriculture and Veterinary Medicine, Al-Qassim University, Buraydah, Al-Qassim Province, Saudi Arabia; 2 Department of Biological Sciences, Kent State University, Kent, OH, 44242, US; 3 School of Biomedical Sciences, Kent State University, Kent, OH, 44242, US; 4 Department of Natural Sciences, Malone University, Canton, OH, 44709, US.; 5 Lerner Research Institute, Cleveland Clinic, Cleveland, OH, 44195, US.

## Abstract

Melanocortin 4 receptor (MC4R) variants contribute to human obesity, and rats lacking functional MC4R (*Mc4r*^*K314X/K314X*^) are obese. We investigated the hypothesis that low energy expenditure (EE) and physical activity contribute to this obese phenotype in male rats, and determined whether lack of functional MC4R conferred protection from weight loss during 50% calorie restriction. Though *Mc4r*^*K314X/K314X*^ rats showed low brown adipose *Ucp1* expression and were less physically active than rats heterozygous for the mutation (*Mc4r*^+*/K314X*^) or wild-type (*Mc4r*^+*/*+^) rats, we found no evidence of lowered EE in *Mc4r*^*K314X/K314X*^ rats once body weight was taken into account using covariance. *Mc4r*^*K314X/K314X*^ rats had a significantly higher respiratory exchange ratio. Compared to *Mc4r*^+*/*+^ rats, *Mc4r*^*K314X/K314X*^ and *Mc4r*^+*/K314X*^ rats lost less lean mass during calorie restriction, and less body mass when baseline weight was accounted for. Limited regional overexpression of *Mc3r* was found in the hypothalamus. Although lower physical activity levels in rats with nonfunctional MC4R did not result in lower total EE during free-fed conditions, rats lacking one or two functional copies of *Mc4r* showed conservation of mass, particularly lean mass, during energy restriction. This suggests that variants affecting MC4R function may contribute to individual differences in the metabolic response to food restriction.

The function of the leptin-melanocortin system is essential to maintaining energy balance homeostasis^1^. The importance of the brain melanocortin system, particularly melanocortin 4 receptor (MC4R), is revealed by disruptions in this system which alter energy balance^2^. Mutations or nonfunctional MC4R result in profound obesity in humans as well as laboratory animals^1^,^2^,^3^, and a large number of variants in *MC4R* account for a notable proportion of variance in adiposity in human polygenic obesity^3^. MC4R functions in the brain to influence appetite and impact physical activity^4^,^5^,^6^. Thus, is it possible that altered physical activity or energy expenditure contribute to the obese phenotype associated with altered MC4R function. Though the specific contribution of diminished physical activity to the rise in obesity has been the cause of some contention, it is evident that lean people show elevated physical activity^7^,^8^. Like obesity, physical activity is highly heritable^9^, and a wealth of potential genes and neural pathways may link genetic background to daily physical activity levels. The brain melanocortin system, and MC4R in particular, appears to make an important contribution to individual differences in both obesity propensity and physical activity^2^,^3^,^10^.

The development of a rat lacking functional MC4R (*Mc4r*^*K314X/K314X*^) has allowed for the investigation of the function of this receptor in energy balance and behavior^4^,^6^,^11^,^12^,^13^,^14^,^15^. Rats lacking functional MC4R are hyperphagic, have higher body weight and adiposity, and have higher levels of circulating leptin and insulin^4^,^12^,^13^,^14^. These rats are also not hypertensive despite their obesity, consistent with lower sympathetic tone^15^,^16^. Behaviorally, these rats show preference for a high-fat diet, as well as a lowered behavioral response to a mixed melanocortin receptor agonist^4^. Though lower levels of physical activity were noted in most cases^4^, the potential contribution of physical activity and energy expenditure to the obese phenotype in the MC4R-deficient rats has not been directly assessed. Here, we measured 24-hr energy expenditure as well as long-term physical activity in male rats homozygous for the non-functional MC4R (*Mc4r*^*K314X/K314X*^), as well as rats with one (*Mc4r*^+*/K314X*^) or two (*Mc4r*^+*/*+^) functional copies of the *Mc4r* gene. Uncoupling protein 1 (*Ucp1*) expression in brown adipose tissue (BAT) was measured, along with central regulators of energy balance and expression levels of *Mc3r* and *Mc5r* in specific hypothalamic nuclei. Lastly, because obesity is linked to inflammatory processes, and melanocortins may counteract inflammation^17^,^18^,^19^, circulating pro- and anti-inflammatory cytokines were measured.

## Results

### MC4R-deficient male rats show greatly enhanced adiposity, elevated RER, and low physical activity levels, but without suppressed overall energy expenditure

**Figure 1:**
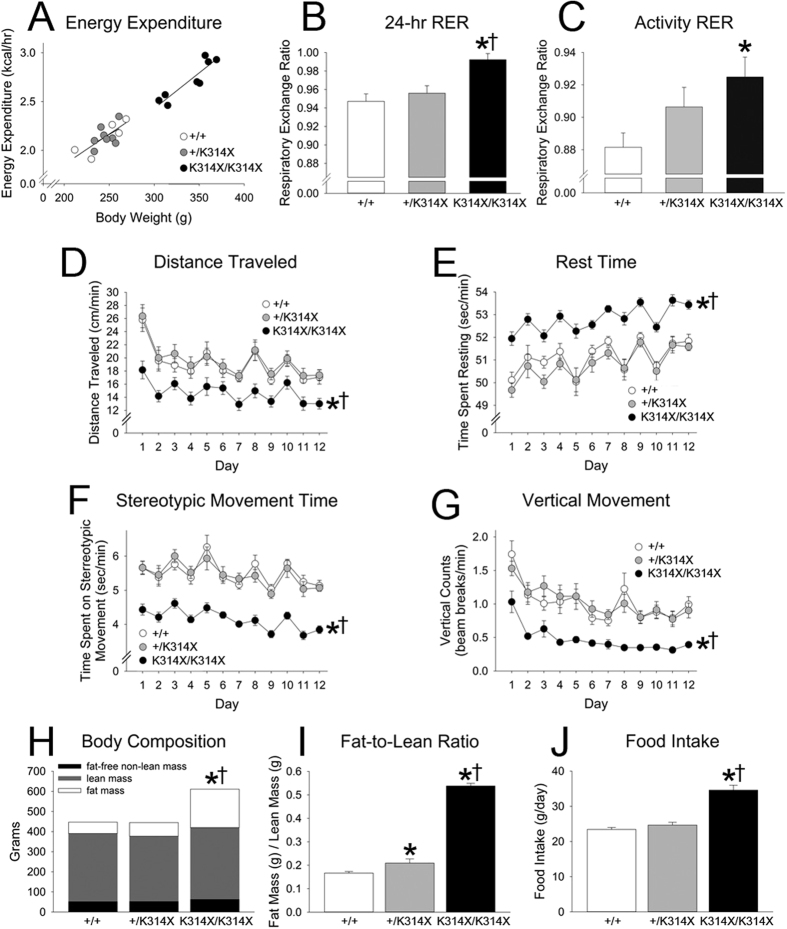
Effects of *Mc4r*^*K314X*^ mutation on energy expenditure, physical activity, body composition, and food intake in male rats. (**A**) When body weight is taken into account using covariance, no difference is seen between genotypes in energy expenditure. (**B**) Respiratory exchange ratio (RER, VCO_2_/VO_2_), an indicator of substrate use, was significantly higher in rats lacking functional MC4R (*Mc4r*^*K314X/K314X*^) compared to wild-type (*Mc4r*^+*/*+^) rats or rats heterozygous for the allele (*Mc4r*^+*/K314X*^) during 24-hr calorimetry. (**C**) During the first 10 min of controlled treadmill activity after short-term food restriction, RER of *Mc4r*^*K314X/K314X*^ rats was higher than *Mc4r*^+*/*+^ rats’ RER, indicating a lower reliance on lipids compared to *Mc4r*^+*/*+^ rats. Over the course of 12 days in activity monitors, *Mc4r*^*K314X/K314X*^ rats (n = 8) showed consistently suppressed physical activity, including (**D**) distance traveled, (**E**) time spent resting, (**F**) stereotypic movements (e.g., grooming), and (**G**) vertical movements (e.g., rearing). (**H**) *Mc4r*^*K314X/K314X*^ rats were significantly heavier than either *Mc4r*^+*/*+^ (n = 8) or *Mc4r*^+*/K314X*^ rats (n = 8), primarily because of fat mass. (**I**) While *Mc4r*^+*/K314X*^ and *Mc4r*^+*/*+^ rats did not differ in body weight, *Mc4r*^+*/K314X*^ rats had a moderately but significantly greater fat-to-lean ratio. (**J**) *Mc4r*^*K314X/K314X*^ rats showed greater daily energy intake; *Mc4r*^+*/K314X*^ and *Mc4r*^+*/*+^ rats did not differ from each other in food intake. *significantly different from *Mc4r*^+*/*+^; ^†^significantly different from *Mc4r*^+*/K314X*^; p < 0.05. For all calorimetry, n = 8 *Mc4r*^*K314X/K314X*^, n = 8 *Mc4r*^+*/K314X*^, n = 6 *Mc4r*^+*/*+^; For all other measures, n = 8 *Mc4r*^*K314X/K314X*^, n = 8 *Mc4r*^+*/K314X*^, n = 8 *Mc4r*^+*/*+^.

Table 1 details body composition at the time of calorimetry. At 7–8 weeks of age, *Mc4r*^*K314X/K314X*^ rats were universally obese, with no overlap in body weight with *Mc4r*^+*/K314X*^ or *Mc4r*^+*/*+^ rats of the same age and sex. The higher body weight in *Mc4r*^*K314X/K314X*^ rats was primarily due to additional fat accumulation (Fig. 1, Table 1).

At the time of calorimetry, *Mc4r*^*K314X/K314X*^ were significantly larger and expended more energy (kcal/hr) than either *Mc4r*^+*/*+^ or *Mc4r*^+*/K314X*^ rats, which did not significantly differ from each other in weight or 24-hr EE (Table 1). As shown in Fig. 1, 24-hr EE did not differ between genotypes when either total body weight or lean mass (each of which accounted for a significant amount of variance in EE) was taken into account as a covariate. As total EE was highest in the *Mc4r*^*K314X/K314X*^ rats, calculated VO_2_ (ml/kg/hr) was significantly lower, also due to the much larger body mass of these rats (Table 1). RER, an indirect measure of metabolic fuel selection, was significantly different between genotypes, with *Mc4r*^*K314X/K314X*^ rats having significantly higher RER than both *Mc4r*^+*/*+^ and *Mc4r*^+*/K314X*^ rats (Fig. 1B). When activity EE was measured using treadmill walking (Table 1), EE was linear within the body weight range of the rats, consistent with the energy cost of light activities in humans^20^. No treadmill-EE variable was significantly different once body weight was taken into account as a covariate, indicating no significant difference in energy cost of locomotion. As shown in Table 1, there were trends (0.10 > p > 0.05) toward higher RER in both *Mc4r*^*K314X/K314X*^ and *Mc4r*^+*/K314X*^ compared to *Mc4r*^+*/*+^ rats during both resting (the last 90 min) and in activity; during the first 10 min of activity, RER was significantly lower in *Mc4r*^+*/*+^ rats compared to *Mc4r*^*K314X/K314X*^ rats (Fig. 1C), and RER was significantly lower in *Mc4r*^+*/*+^ rats compared to all other rats combined over the course of the 30 min of treadmill walking.

At the time of long-term activity monitoring, all rats had gained weight, and body weight and adiposity remained significantly higher in *Mc4r*^*K314X/K314X*^. As shown in Fig. 1I, in *Mc4r*^+*/K314X*^ rats, fat contributed significantly more, and lean mass significantly less, to their total mass, resulting in a greater fat-to-lean mass ratio, despite body weights similar to *Mc4r*^+*/*+^ rats. *Mc4r*^*K314X/K314X*^ rats were hyperphagic, eating significantly more than either *Mc4r*^+*/K314X*^ or *Mc4r*^+*/*+^ rats; water intake similarly differed among groups, generally mirroring food intake (Table 2). *Mc4r*^*K314X/K314X*^ rats showed lower activity levels in every dimension measured except for the number of bursts of vertical activity (p = 0.072). *Mc4r*^*K314X/K314X*^ rats spent more time resting; covered less distance each day; spent less time in ambulatory, vertical movement, and stereotypic movement; had fewer beam-break counts in horizontal, ambulatory, and vertical activity; had fewer bursts of stereotypic movement; and showed fewer clockwise and counterclockwise rotations (Fig. 1, Table 2). When long-term physical activity was analyzed over time (Fig. 1D–G), activity on the first day of measurement was high due to the novel environment, with some subsequent day-to-day variance thereafter that affected all rats similarly. *Mc4r*^+*/*+^ rats were not more active than *Mc4r*^+*/K314X*^ in any dimension of activity measured. The correlation between body weight and distance traveled within *Mc4r*^*K314X/K314X*^ rats was r = 0.67 (p = 0.072). Lastly, for the *Mc4r*^*K314X/K314X*^ rats with two consecutive measurements of 12-day activity (n = 6, correlation r = 0.95), body weight increased (by 117 g; p < 0.01) but there was no significant change in physical activity in these rats over time (−0.54 cm/min).

### Partial or full MC4R deficiency is protective during food restriction

**Figure 2:**
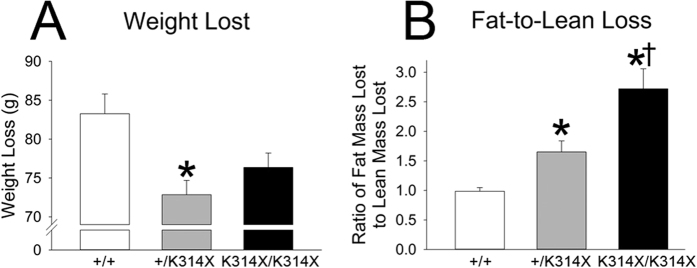
Weight loss during 50% food restriction in male rats homozygous (*Mc4r*^*K314X/K314X*^) or heterozygous (*Mc4r*^+*/K314X*^) for the non-functional melanocortin 4 receptor (MC4R), compared to wild-type rats (*Mc4r*^+*/*+^). (**A**) *Mc4r*^+*/*+^ rats lost more weight compared to *Mc4r*^+*/K314X*^ rats; these groups did not show differences in baseline food intake or body weight. (**B**) The relative loss of fat mass differed according to genotype, where most of the weight lost in *Mc4r*^*K314X/K314X*^ was from fat mass, and nearly half of the weight lost in *Mc4r*^+*/*+^ was from lean mass. N = 8/group. *significantly different from *Mc4r*^+*/*+^; ^†^significantly different from *Mc4r*^+*/K314X*^; p < 0.05.

As shown in Fig. 2, genotype affected weight loss. Planned comparisons revealed *Mc4r*^+*/*+^ rats lost significantly more weight (in grams) than *Mc4r*^+*/K314X*^ rats (p = 0.0026); these groups showed similar starting weights and food intake. Weight loss as percent of baseline body weight was significantly different between each genotype, with *Mc4r*^+*/*+^ rats losing a greater proportion of their baseline weight than *Mc4r*^+*/K314X*^, and *Mc4r*^+*/K314X*^ losing a greater proportion than *Mc4r*^*K314X/K314X*^. When differences in starting weight were taken into account using baseline body weight (g) as a covariate, a significant effect of genotype was found on weight loss (p < 0.01), with significant differences between each genotype. There were no significant correlations between weight gain (g) during baseline feeding and weight loss (g) during CR within genotype or overall.

Loss of fat and lean mass from 50% CR differed between genotypes (Fig. 2B, Table 3). *Mc4r*^*K314X/K314X*^ rats lost significantly more grams of fat than either *Mc4r*^+*/*+^ or *Mc4r*^+*/K314X*^, and *Mc4r*^+*/*+^ rats lost significantly more lean mass than either *Mc4r*^+*/K314X*^ or *Mc4r*^*K314X/K314X*^ rats; *Mc4r*^+*/K314X*^ rats also lost more lean mass than *Mc4r*^*K314X/K314X*^ rats. Baseline adiposity (grams fat) and change in body mass did not significantly correlate with each other (r = 0.12) except when *Mc4r*^*K314X/K314X*^ rats were analyzed alone, where *Mc4r*^*K314X/K314X*^ with more initial body fat lost more weight (r  = −0.71; p < 0.05). After a 2-week *ad libitum*-feeding recovery period, regain of body composition favored lean mass (Table 3); rats regained all or nearly all of their lean mass, but not fat mass.

### Cytokine levels, and gene expression in brain, muscle, and white and brown adipose tissue

Plasma leptin was significantly different between genotypes, with *Mc4r*^*K314X/K314X*^ rats having significantly higher leptin levels than both other genotypes (Table 4). Serum levels of the inflammatory cytokines interleukin (IL)-1β, tumor necrosis factor-α (TNF-α), and IL-6, as well as anti-inflammatory IL-10, did not significantly differ between *Mc4r*^+*/*+^, *Mc4r*^+*/K314X*^, and *Mc4r*^*K314X/K314X*^ rats. Epididymal white adipose (WAT) TNF-α did not differ between groups. There was high intragroup variation in serum levels of IL-1β and IL-10 and in WAT TNF-α. There was no group difference in the expression of lipogenic genes peroxisome proliferator-activated receptor γ (*Pparγ*)^21^ or sterol regulatory element-binding protein 1 (*Srebf1*)^22^,^23^ in WAT. As a percent of *Mc4r*^+*/*+^, *Pparγ*: *Mc4r*^+*/*+^ 100±28, *Mc4r*^+*/K314X*^ 115 ± 23, *Mc4r*^*K314X/K314X*^ 119 ± 23; *Srebf1*: *Mc4r*^+*/*+^ 100 ± 21, *Mc4r*^+*/K314X*^ 148 ± 22, *Mc4r*^*K314X/K314X*^ 100 ± 24.

*Mc4r*^*K314X/K314X*^ rats showed region- and subtype-specific differences in MC receptor expression in the hypothalamus (Table 4). Higher levels of *Mc3r* mRNA were found in the paraventricular nucleus (PVN); other regions showed non-significant trends in the same direction. For brain derived neurotrophic factor (BDNF) mRNA, significantly higher levels were seen in the PVN of *Mc4r*^+*/*+^ and *Mc4r*^*K314X/K314X*^ rats compared to *Mc4r*^+*/K314X*^ rats, with no significant difference in BDNF in the ventromedial nucleus of the hypothalamus (VMH).

Lastly, BAT *Ucp1* expression was significantly lower in *Mc4r*^*K314X/K314X*^ rats (as percent of *Mc4r*^+*/*+^: 22 ± 11) than either *Mc4r*^+*/K314X*^ rats (96 ± 21) or *Mc4r*^+*/*+^ rats (100 ± 22). The low amount of BAT found in *Mc4r*^*K314X/K314X*^ rats prevented Western blot comparison of UCP1 protein. In order to confirm the identity of the BAT, expression of genes primarily expressed in WAT and not BAT^24^ were measured. Expression was significantly higher in WAT than BAT for both inhibin beta B gene (*Inhbb*; 2^−ΔCT^: BAT 0.0027 ± 0.0008, WAT 0.0321 ± 0.0125) and *Hoxc8*^24^ (2^−ΔCT^: BAT 0.0019 ± 0.0003, WAT 0.1326 ± 0.0182); no genotype differences were found within WAT or BAT for *Inhbb* or *Hocx8*.

## Discussion

We investigated the potential contribution of total, whole-body EE and physical activity to the obese phenotype seen in rats lacking functional MC4R, and the impact of food restriction on adiposity in these rats. The *Mc4r*^*K314X/K314X*^ rats were hyperphagic and showed very low levels of daily physical activity, but no differences in EE were found once body weight and composition were taken into account (Fig. 1, Table 1). Considering their very large size and therefore higher energy budget, reflected in their higher absolute EE (Fig. 1A), *Mc4r*^*K314X/K314X*^ rats showed remarkable conservation of body mass—especially lean mass—during food restriction (Fig. 2A). Rats heterozygous for the altered *Mc4r* gene appeared nearly indistinguishable from the *Mc4r*^+*/*+^ rats with two exceptions. First, compared to *Mc4r*^+*/*+^ rats, *Mc4r*^+*/K314X*^ rats had a significantly higher fat-to-lean mass ratio without a significant difference in body weight (Fig. 1H,I); this is similar to other studies, though small differences in food intake or body weight between *Mc4r*^+*/*+^ and *Mc4r*^+*/K314X*^ rats have been reported^4^,^12^. Second, the *Mc4r*^+*/K314X*^ rats lost less weight than *Mc4r*^+*/*+^ rats during equivalent calorie restriction; thus, there is a meaningful impact of heterozygosity of *Mc4r*^*K314X*^ that is not apparent when examining body weight during baseline conditions. Animals with reduced MC4R function would likely be well suited to conditions of food shortage.

Not surprising given the major influence of body size in determining EE, larger rats expended more calories, and the *Mc4r*^*K314X/K314X*^ rats as a group showed elevated overall EE (Table 1). Once body mass was taken into consideration using regression-based adjustment for body weight^25^, however, there were no group differences in 24-hr EE (Fig. 1A). This was unexpected given that the obesity and extreme adiposity seen in *Mc4r*^*K314X/K314X*^ rats is accompanied by elevated food intake and feed efficiency, with no change in feed conversion efficiency^4^,^12^. Similar to 24-hr EE, activity-induced EE was unaffected by genotype (Table 1). We found low levels of UCP1 mRNA in BAT of *Mc4r*^*K314X/K314X*^ rats, which also implicated low EE as a potential contributor to obesity in these rats. As the rats were housed under thermoneutral conditions, we did not assess the potential contribution of BAT thermogenesis to the obese phenotype of *Mc4r*^*K314X/K314X*^ rats, for example during cold stress or overfeeding.

Calorimetry revealed an exceptionally elevated RER in the *Mc4r*^*K314X/K314X*^ (Fig. 1B). This is in line with reports of humans with loss-of-function mutations of this receptor^26^, and in *Mc4r*-null mutant mice, though this was dependent on diet^27^. High RER is associated with a reliance on carbohydrates for fuel and low fat oxidation (net lipogenesis). Normally, assigning causality to altered RER is difficult as acute factors such as activity and food intake will impact RER. There was a trend toward elevated RER in both the *Mc4r*^*K314X/K314X*^ and *Mc4r*^+*/K314X*^ rats even during (2 hr) food restriction, and significantly lower RER in *Mc4r*^+*/*+^ during treadmill activity (Table 1, Fig. 1C). This implicates differences in fat oxidation during activity, consistent with the known role of MC4R in regulating autonomic outflow at the levels of the hypothalamus and preganglionic neurons^28^,^29^. MC4R stimulates sympathetic and/or decreases parasympathetic outflow to multiple systems including white adipose tissue, where MC receptor activation is known to induce lipolysis^27^,^30^,^31^. This corresponds to the high adiposity in *Mc4r*^*K314X/K314X*^ rats and the moderately elevated adiposity of *Mc4r*^+*/K314X*^ rats, and is also in line with the association between high respiratory quotient and weight gain in humans^32^. Though increased mRNA expression of lipogenic enzymes is seen after central inhibition of melanocortin receptors with SHU9119^26^, we found no difference between genotypes in their expression of lipogenic genes *Pparγ* or *Srebf1* in WAT. Lastly, the significant loss of adipose tissue during food restriction in *Mc4r*^*K314X/K314X*^ rats (Fig. 2B) suggests that the ability of negative energy balance to induce fat mobilization was not impaired in either *Mc4r*^*K314X/K314X*^ or *Mc4r*^+*/K314X*^ rats.

*Mc4r*^*K314X/K314X*^ rats were not markedly hypometabolic despite the lower levels of physical activity seen in these rats during calorimetry (Fig. 1, Tables 1 and 2) as well as during long-term, 12-day activity measurements (Fig. 1, Table 2). *Mc4r*^*K314X/K314X*^ rats showed low physical activity in nearly every dimension of activity measured (Fig. 1), consistent with evidence from mice lacking this receptor^33^. It is unlikely that low daily activity levels are secondary to the elevated body mass in the *Mc4r*^*K314X/K314X*^ rats because, along with lower ambulatory and vertical activity, these rats showed less stereotypical activity (e.g., grooming), which is unlikely to be affected by body size. Distance traveled did not significantly correlate with body weight (non-significant trend in the opposite direction). Additionally, when activity was measured twice consecutively in 6 *Mc4r*^*K314X/K314X*^ rats, physical activity did not significantly decrease over time despite a substantial gain in body weight (117 g). Thus, it is likely that low physical activity is an integral part of the *Mc4r*^*K314X/K314X*^ phenotype, consistent with other aspects of behavior in *Mc4r*^*K314X/K314X*^ rats, such as low levels of grooming behavior^4^,^6^. Because body size contributes relatively more to activity EE^34^ than to basal or resting EE, it is possible that the *Mc4r*^*K314X/K314X*^ rats’ low activity levels were offset by their much larger body weights, augmenting their activity EE. The suppressed activity seen in *Mc4r*^*K314X/K314X*^ rats is consistent with the known role of melanocortin receptors in modulating physical activity^10^,^33^,^35^. MC4R exerts some of its actions on behavior through peptides including BDNF^36^; our qPCR showed that *Mc4r*^*K314X/K314X*^ rats did not have suppressed *Bdnf* expression in the PVN or VMH, although PVN *Bdnf* was reduced in *Mc4r*^+*/K314X*^ rats (Table 4). Elevated expression of *Mc3r* was seen in the PVN of *Mc4r*^*K314X/K314X*^ rats, reflecting trends in *Mc3r* but not *Mc5r* expression in most other brain regions (Table 4), consistent with elevated hypothalamic *Mc3r* expression previously reported^4^. Lastly, rats heterozygous for the mutation were not less physically active than *Mc4r*^+*/*+^ rats in any measure of activity.

Although *Mc4r*^+*/K314X*^ and *Mc4r*^+*/*+^ rats started out at very similar body weights and energy needs, and indistinguishable physical activity levels (Fig. 1), *Mc4r*^+*/*+^ rats lost significantly more grams of body weight than *Mc4r*^+*/K314X*^ rats (Fig. 2A). This suggests the possibility that differential MC4R function may lead to individual differences in response to energy restriction, for example though differential adaptive thermogenic response, though additional calorimetric measurements would be needed to explicitly test this hypothesis. Given the *Mc4r*^*K314X/K314X*^ rats’ very large size and high energy budget (Fig. 1A,J), and greater kcal deficit relative to *Mc4r*^+*/K314X*^ and *Mc4r*^+*/*+^, it would be predicted that *Mc4r*^*K314X/K314X*^ rats would be prone to lose more weight than their smaller counterparts. Contrary to this, *Mc4r*^*K314X/K314X*^ rats did not lose more grams of body weight than the other genotypes (Fig. 2). *Mc4r*^*K314X/K314X*^ lost a lower proportion of their baseline body weight, however, this calculation may not correctly adjust for baseline body weight^37^. Covariate analysis revealed that *Mc4r*^*K314X/K314X*^ rats lost less weight compared to *Mc4r*^+*/K314X*^ or *Mc4r*^*K314X/K314X*^ than would be predicted by variance in baseline body weight. Correlation analysis showed that this was not reflected at the individual level—within group or across all rats, individual rats that showed more weight gain during baseline food-intake calculations did not lose significantly less (or more) weight during CR, indicating that group differences in weight loss between genotypes were not secondary to potential bias in food allocation calculations for CR.

When considering the change in body composition during CR, lean mass was lost along with fat mass, but *Mc4r*^*K314X/K314X*^ rats lost significantly more fat mass than the other genotypes, while *Mc4r*^+*/*+^ rats lost more lean mass than the other genotypes (Fig. 2, Table 3). In other words, loss of MC4R function was associated with a more appropriate or adaptive response to caloric deficit, where fat stores are utilized while conserving lean mass. Baseline adiposity may contribute to the ability to maintain lean mass during CR^38^, however, the large difference between *Mc4r*^+*/*+^ and *Mc4r*^+*/K314X*^ in the ability to conserve lean mass, compared to their moderate (but significant) difference in baseline adiposity, strongly supports the assertion that MC4R modulates fat mobilization during energy deficit. The ability of *Mc4r*^*K314X/K314X*^ rats to conserve body weight and lean mass suggests that, relative to *Mc4r*^+*/*+^, these rats would likely have increased fitness in an environment where food is scarce or insecure. Moreover, rats heterozygous for the mutant allele showed an intermediate phenotype in their conservation of weight and lean mass, despite their similarity to *Mc4r*^+*/*+^ rats in nearly every other respect. It is conceivable that even a single copy of the mutant allele could confer a survival advantage during food shortages, promoting maintenance of the mutant allele in the rat population; the consistent association between *MC4R* mutations and variants with human obesity supports this idea^2^,^3^,^39^. Lastly, unlike weight and fat loss, recovery from CR was roughly equivalent among genotypes (Table 3), except that *Mc4r*^+*/*+^ rats initially showed faster weight regain.

Because of the well-known connection between the melanocortin system and inflammation^40^,^41^, we investigated the potential impact of MC4R genotype on pro- and anti-inflammatory cytokines. Though melanocortins and the associated receptors have been shown to be anti-inflammatory and alter cytokine levels^42^, neither *Mc4r*^*K314X/K314X*^ nor *Mc4r*^+*/K314X*^ rats showed any significant differences in the either the pro- or anti-inflammatory cytokines measured here (Table 4). The anti-inflammatory effects of α-MSH may be mediated through another melanocortin receptor, or the potential effects of MC4R may have been obscured due to developmental compensation^4^ (Table 4). Leptin was significantly higher in rats lacking both *Mc4r* alleles, consistent with previous reports;^4^,^13^,^16^ this is likely secondary to their greater fat mass.

In summary, though *Mc4r*^*K314X/K314X*^ rats showed lower levels of physical activity, this did not lead to a detectable suppression of daily EE, potentially because of the greater energy requirements to move the larger mass of the *Mc4r*^*K314X/K314X*^ rats. This implicates a larger role of hyperphagia in the obese phenotype seen in rats lacking functional MC4R^4^,^12^, and potentially in human obesity seen with altered MC4R. During food restriction, on the other hand, a clear energetic phenotype of these rats emerged, with the *Mc4r*^*K314X/K314X*^ rats displaying enhanced conservation of mass, especially lean mass; though rats with impaired MC4R function lost less weight, more of the weight loss was attributable to fat loss. Despite the similarity between *Mc4r*^+*/*+^ rats and rats with only one copy of the functional *Mc4r* gene under free-fed conditions, *Mc4r*^+*/K314X*^ rats were also better able to conserve mass during calorie restriction. The ability of rats with compromised MC4R function to appropriately mobilize fat and retain lean mass during energy restriction suggests that their altered MC4R function may confer a selective advantage during energy shortage.

## Methods

### Animals

Adult male *Mc4r*^*K314X/K314X*^, *Mc4r*^+*/K314X*^, and *Mc4r*^+*/*+^ rats (n = 8/group)^4^,^6^,^12^,^16^,^43^ were obtained from Taconic. Rats were housed on a 12:12 light:dark cycle with lights-on at 0700 (EST) and provided *ad libitum* access to water and (before calorie restriction) food (5P00 MRH 3000, T.R. Last Co. Inc). Animal protocols were approved by the Kent State University Institutional Animal Care and Use Committee, and all methods were performed in accordance with the relevant guidelines and regulations. Body weights were measured using a Denver Instruments XP-1500 balance. Body composition was measured using an EchoMRI-700 (EchoMRI, Houston, TX).

### Calorimetry

Rats were measured for body composition and acclimated for 2 days before 24-hr energy expenditure (EE) was measured using a 4-chamber Oxymax FAST system (Columbus Instruments) as described previously^34^. For analysis of 24-hr EE, 3 *Mc4r*^+*/*+^ rats were excluded from only this analysis because they weighed significantly more than the other *Mc4r*^+*/*+^ due to a 3-week age difference compared to all other rats. Resting EE (2 hrs without food) and EE during low levels of activity (walking) were measured using a treadmill as described previously^34^,^44^; 1 *Mc4r*^+*/*+^ and 2 *Mc4r*^+*/K314X*^ rats were noncompliant and excluded from the analysis.

### Long-term physical activity

To allow for a more detailed analysis of different dimensions of physical activity in the X, Y, and Z axes, long-term physical activity levels were assessed over 12 days in 16 × 16-inch chambers using an Opto-Varimex 4 Auto-Track system (Columbus Instruments), as described previously^45^. Activity was measured in two cohorts, first in 16 rats (6 *Mc4r*^*K314X/K314X*^, 4 *Mc4r*^+*/K314X*^, and 6 *Mc4r*^+*/*+^ randomly assigned to 16 chambers). The second cohort contained the remaining 8 rats along with 8 rats from the first cohort (6 *Mc4r*^*K314X/K314X*^, 2 *Mc4r*^+*/*+^); comparison of data from rats measured twice confirmed consistency of measurement between the cohorts, so duplicate measurements were averaged. Mean age at beginning of activity measurement was 84 days for *Mc4r*^+*/*+^, 82 days for *Mc4r*^+*/K314X*^, and 84 days for *Mc4r*^*K314X/K314X*^.

### Weight loss during calorie restriction

Daily food intake was measured in each rat for 7 days, and the average food intake was calculated omitting the days of highest and lowest food intake for each rat. Rats were then fed 50% of their daily *ad libitum* food intake for 21 days (50% CR). Body composition was measured 2 days before the onset of 50% CR as well as on the 21st day of CR. Body weight was measured daily before food was given at 1200 hr ± 1 hr. After the conclusion of 50% CR, rats were again given *ad libitum* access to food; daily measurements continued for one week then every 2–3 days for another week; body composition was measured after 14 days of *ad libitum* feeding.

### Cytokines and qPCR analysis

After the conclusion of the study, rats were euthanized by rapid decapitation; brain, BAT, WAT, and serum were collected. Serum levels of inflammatory cytokines IL-1β, TNF-α, and IL-6, anti-inflammatory cytokine IL-10, and leptin were determined by the CTSC Bioanalyte Core Center of Case Western Reserve University using the Luminex xMAP multiplexing ELISA system, which obtains data from multiple cytokines from each individual sample, read and analyzed with the Luminex 200™ and xPONENT^®^ software (LuminexCorporation, EMD Millipore). WAT TNF-α levels were analyzed using an enzyme-linked immunosorbent assay (ELISA; Abcam) in accordance with manufacturer instructions. Tissue was minced, then homogenized after adding HNTG lysis buffer (50 mM HEPES, 150 mM NaCl, 10% glycerol, and 1% Triton-X 100, pH 7.5). Samples were rotated for 30 minutes at 4 °C, followed by centrifugation (18,000 × g, 4 °C) for 20 minutes; supernatant protein content was measured using the Bradford assay and tissue samples were equalized according to protein content.

Referencing an atlas^46^, brains were sectioned at 40 μm on a cryostat (Leica CM1950) and hypothalamic nuclei were dissected from frozen tissue by micropunch. 1 mm and 2 mm microdissection tools were used as described previously^10^,^47^ to obtain samples of the arcuate nucleus (ARC), PVN, perifornical lateral hypothalamic area (PeFLH), VMH, and dorsomedial hypothalamus (DMH) (*Mc4r*^*K314X/K314X*^, n = 5; *Mc4r*^+*/K314X*^, n = 6; *Mc4r*^+*/*+^, n = 4). Tissue samples were homogenized in TRI reagent (Ambion), incubated with 100 μL of bromochloropropane (Molecular Research Center, Cincinnati, Ohio), and centrifuged (12,000 g for 10 minutes at 4 °C). 150 μL of 100% ethanol was added to the extracted supernatant, then an Ambion Ribopure kit was used to separate the RNA. RNA concentration was measured in a NanoDrop ND-1000 spectrophotometer. Quantitative real-time PCR (qPCR) was performed using Brilliant III Ultra-Fast QPCR Master Mix (Agilent) and Taqman probes. All samples were run in triplicate on Strategene Mx3005 P (Agilent) as described previously^10^. qPCR was performed with the same protocols in WAT and BAT to determine expression of BAT *Ucp1*, the WAT markers *Inhbb* and *Hoxc8* in both WAT and BAT, and WAT lipogenic modulators *Pparγ* and *Srebf1*. The control gene for all qPCR assays was GAPDH; the Comparative Ct method (ΔΔCt) method was used to calculate relative expression. The mean of *Mc4r*^+*/*+^ rats’ ΔΔCt values was defined as 100%, and each animal’s gene expression was calculated as a percentage of that mean.

### Statistical analysis

Energy expenditure data (kcal/hr) were analyzed using analysis of covariance (ANCOVA), with body weight and lean mass as covariates in separate analyses. Activity and respiratory exchange ratio (RER) data collected during calorimetry were analyzed using a 1-way analysis of variance (ANOVA), using LSD tests for post-hoc comparisons. Treadmill resting and walking (30 min at 7 meters/min) gas exchange data were calculated and analyzed using ANCOVA. A mixed repeated measures (spit-plot) ANOVA was used to analyze the long-term physical activity data and to compare body weight and composition before vs. after 50% CR, with planned (*a priori*) comparisons (with Bonferroni correction, p < 0.017 for 3 groups) of absolute weight loss between genotype; weight loss (in g) was also compared between genotypes using ANCOVA with baseline body weight as the covariate. A single missing body weight value during CR was estimated using imputation for the ANOVAs, but not used in post-hoc or planned analyses. Pearson’s correlation coefficient (r) was used to address alternative hypotheses regarding weight loss and baseline adiposity. A 1-way ANOVA was used to analyze change in body composition, expression of brain and muscle genes and proteins, and plasma cytokines. For cytokines, statistical outliers were identified using the outlier labeling rule (using 2.2*(75^th^-25^th^ percentile)) to determine cut-off values above and below the 25^th^ and 75^th^ percentiles). For qPCR, mean and variance values were calculated and 2-tailed t-tests were used to compare groups. A significance cut-off of *p* < 0.05 was used.

## Additional Information

**How to cite this article**: Almundarij, T. I. *et al*. Physical Activity, Energy Expenditure, and Defense of Body Weight in Melanocortin 4 Receptor-Deficient Male Rats. *Sci. Rep*. **6**, 37435; doi: 10.1038/srep37435 (2016).

**Publisher’s note:** Springer Nature remains neutral with regard to jurisdictional claims in published maps and institutional affiliations.

## Figures and Tables

**Table 1:** Body composition and calorimetry measures from measurement of 24-hr energy expenditure (EE), and activity-EE (treadmill test); Mean (SEM).

Genotype	Body Weight (g)	Lean Mass (g)	Fat Mass (g)	% Fat	% Lean	VO_2_ (ml/kg/hr)	VCO_2_ (ml/kg/hr)	EE (kcal/hr)	Horizontal Activity (counts/min)	Ambulatory Activity (counts/min)	Vertical Activity (counts/min)	Treadmill EE (kcal/hr)			Treadmill RER	
												Resting EE	Total activity EE	Activity EE-resting EE	Last 90 min of rest	Total 30 min of activity
*Mc4r*^*K314X/K314X*^	339.55^*,†^ (8.77)	223.63^*,†^ (5.56)	65.48^*,†^ (3.86)	20.04^*,†^ (0.78)	68.80^*,†^ (0.79)	1588^*,†^ (16.44)	1580 (21.36)	2.72^*,†^ (0.07)	5.01^*,†^ (0.21)	2.31* (0.13)	0.21^*,†^ (0.02)	2.37 (0.06)	5.67 (0.29)	3.30 (0.27)	0.87 (0.01)	0.90 (0.01)
*Mc4r*^+*/K314X*^	245.24 (7.78)	183.51 (3.16)	25.82 (1.52)	10.77 (0.64)	76.53 (1.07)	1743 (26.85)	1671 (32.79)	2.14 (0.04)	6.12 (0.26)	2.68 (0.13)	0.35 (0.02)	1.93 (0.05)	4.52 (0.16)	2.59 (0.17)	0.87 (0.82)	0.90 (0.01)
*Mc4r*^+*/*+^	244.88 (8.31)	183.88 (6.36)	24.86 (1.28)	10.67 (0.51)	78.72 (0.34)	1684 (31.15)	1679 (42.12)	2.14 (0.06)	6.72 (0.16)	2.98 (0.11)	0.36 (0.02)	1.97 (0.14)	4.57 (0.18)	2.60 (0.23)	0.82 (0.02)	0.87 (0.01)

Male rats homozygous (*Mc4r*^*K314X/K314X*^, n = 8) or heterozygous (*Mc4r*^+*/K314X*^, n = 8) for non-functional melanocortin 4 receptor (MC4R), or wild-type rats (*Mc4r*^+*/*+^, n = 6). RER, respiratory exchange ratio (VCO_2_/VO_2_).

*significantly different from *Mc4r*^+*/*+^; ^†^significantly different from *Mc4r*^+*/K314X*^; p < 0.05.

**Table 2:** Long-term activity measurements; Mean (SEM).

Genotype	BW (g)	Food Intake (g)	Water intake (ml)	Ambulatory Time (sec/min)	Bursts of stereotypic activity (per min)	Horizontal counts (per min)	Ambulatory counts (per min)	Bursts of vertical activity (per min)	CW rotations (per min)	CCW rotations (per min)	Fat mass (g)	Lean mass (g)
*Mc4r*^*K314X/K314X*^	509.53^*,†^ (10.99)	34.61^*,†^ (1.38)	47.41^*,†^ (2.45)	2.72† (0.1)	2.75^*,†^ ±0.05	16.46^*,†^ (0.95)	9.10^*,†^ (0.68)	0.03 (0.01)	0.27^*,†^ (0.01)	0.26^*,†^ (0.01)	191.07^*,†^ (6.23)	355.49† (10.11)
*Mc4r*^+*/K314X*^	361.13 (12.10)	24.69 (2.15)	36.98 (1.69)	3.34 (0.11)	3.45 ±0.08	23.22 (0.94)	12.83 (0.65)	0.05 (0.01)	0.35 (0.01)	0.35 (0.01)	67.98 (6.34)	323.2 (5.74)
*Mc4r*^+*/*+^	360.88 (15.59)	23.44 (1.54)	35.17 (1.98)	3.04 (0.17)	3.36 ±0.09	24.32 (0.66)	13.49 (0.56)	0.06 (0.01)	0.32 (0.02)	0.32 (0.02)	56.16 (2.04)	337.31 (6.31)

Male rats homozygous (*Mc4r*^*K314X/K314X*^, n = 8) or heterozygous (*Mc4r*^+*/K314X*^, n = 8) for non-functional melanocortin 4 receptor (MC4R), or wild-type rats (*Mc4r*^+*/*+^, n = 8).

CW: clockwise; CCW: counter-clockwise.

*significantly different from *Mc4r*^+*/*+^; ^†^significantly different from *Mc4r*^+*/K314X*^; p < 0.05.

**Table 3:** Body weight and composition in male rats before and after 21 days of 50% calorie restriction (CR), and after 14 days of *ad libitum* recovery from CR; Mean (SEM).

Genotype	Baseline			Calorie restriction			CR-induced change		
	BW (g)	Fat mass (g)	Lean mass (g)	BW (g)	Fat mass (g)	Lean mass (g)	Fat mass (g)	Lean mass (g)	% BW lost
*Mc4r*^*K314X/K314X*^	705.46^*,†^ (22.25)	263.50^*,†^ (10.06)	389.68^*,†^ (13.31)	629.66^*,†^ (17.62)	198.49* (6.53)	364.38^*,†^ (11.69)	−65.01^*,†^ (5.61)	−25.31^*,†^ (2.27)	−10.67^*,†^ (0.46)
*Mc4r*^+*/K314X*^	505.16 (11.70)	103.37* (7.26)	353.32 (5.12)	431.34 (12.54)	51.40* (5.64)	319.66 (7.20)	−51.98 (2.10)	−33.66* (2.87)	−14.96* (0.57)
*Mc4r*^+*/*+^	500.68 (9.79)	80.78 (2.27)	368.45 (7.44)	415.34 (9.16)	33.98 (1.77)	320.21 (6.59)	−46.80 (1.79)	−48.24 (2.21)	−17.06 (0.62)
	Ad libitum-fed recovery from CR			Recovery-induced increase from CR			Recovery-induced increase relative to baseline		
	BW (g)	Fat mass (g)	Lean mass (g)	Increase in BW (g)	Increase in fat mass (g)	Increase in lean mass (g)	Change in BW (g)	Change in fat (g)	Change in lean mass (g)
*Mc4r*^*K314X/K314X*^	708.04^*,†^ (21.56)	233.41^*,†^ (8.01)	401.35^*,†^ (14.65)	78.39 (4.81)	34.93 (2.78)	36.98* (3.22)	2.59 (3.86)	−30.08^*,†^ (3.26)	11.67* (3.01)
*Mc4r*^+*/K314X*^	509.11 (12.26)	84.23* (6.31)	359.94 (6.39)	77.76 (3.28)	32.84 (1.76)	40.27 (2.45)	3.95 (3.31)	−19.14 (2.08)	6.61 (2.16)
*Mc4r*^+*/*+^	498.68 (8.13)	62.86 (1.91)	368.46 (7.44)	83.33 (3.29)	28.89 (1.83)	48.26 (2.64)	−2.00 (4.09)	−17.92 (2.86)	0.02 (1.97)

Male rats homozygous (*Mc4r*^*K314X/K314X*^, n = 8) or heterozygous (*Mc4r*^+*/K314X*^, n = 8) for non-functional melanocortin 4 receptor (MC4R), or wild-type rats (*Mc4r*^+*/*+^, n = 8).

BW: Body weight.

*significantly different from *Mc4r*^+*/*+^; ^†^significantly different from *Mc4r*^+*/K314X*^; p < 0.05.

**Table 4:** Regional mRNA expression of melanocortin receptors 3 (MC3R) and 5 (MC5R), percent expression relative to *Mc4r*^+*/*+^ (100%); circulating and adipose cytokines, Mean (SEM).

Genotype	ARC		PVN			PeFLH		VMH			DMH		Cytokine (pg/ml)						WAT
	MC3R	MC5R	MC3R	MC5R	BDNF	MC3R	MC5R	MC3R	MC5R	BDNF	MC3R	MC5R	Leptin	IL-1α	IL-1β	IL-6	IL-10	TNF-α	TNF-α
*Mc4r*^*K314X/K314X*^	158 (32)	101 (7)	109^*,†^ (2)	108 (3)	102† (3)	104 (2)	102 (5)	149 (8)	97 (7)	120 (9)	129 (11)	125 (14)	11962.00^*,†^ (808.26)	44.58 (6.65)	387.85 (89.10)	33.76 (2.14)	293.89 (84.58)	25.79 (2.90)	349 (29)
*Mc4r*^+*/K314X*^	112 (4)	98 (9)	96 (3)	94 (4)	91* (3)	156 (7)	115 (7)	131 (14)	96 (3)	108 (7)	122 (14)	132 (12)	9843.50 (541.98)	37.31 (2.00)	205.99 (34.16)	35.36 (2.82)	170.06 (30.88)	23.14 (0.64)	383 (48)
*Mc4r*^+*/*+^	100 (3)	100 (6)	100 (4)	100 (4)	100 (1)	100 (6)	100 (1)	100 (12)	100 (10)	100 (7)	100 (5)	100 (5)	9425.75 (534.51)	43.40 (6.30)	370.06 (92.45)	34.73 (3.33)	257.50 (66.48)	26.06 (1.75)	537 (195)

Rats homozygous (*Mc4r*^*K314X/K314X*^, n = 5) or heterozygous (*Mc4r*^+*/K314X*^, n = 6) for non-functional melanocortin 4 receptor (MC4R), or wild-type rats (*Mc4r*^+*/*+^, n = 4).

ARC: arcuate nucleus; PVN: paraventricular nucleus; PeFLH: perifornical lateral hypothalamus; VMH: ventromedial hypothalamus; DMH: dorsomedial hypothalamus; WAT: epididymal white adipose tissue. Cytokines (n = 8/group) interleukins (IL) 1α, 1β, 6, and 10, and tumor necrosis factor- α (TNF- α).

*significantly different from *Mc4r*^+*/*+^; ^†^significantly different from *Mc4r*^+*/K314X*^; p < 0.05.
