# Author Correction: Physical Activity, Energy Expenditure, and Defense of Body Weight in Melanocortin 4 Receptor-Deficient Male Rats

**DOI:** 10.1038/s41598-019-42850-2

**Published:** 2019-04-23

**Authors:** Tariq I. Almundarij, Mark E. Smyers, Addison Spriggs, Lydia A. Heemstra, Lisa Beltz, Eric Dyne, Caitlyn Ridenour, Colleen M. Novak

**Affiliations:** 1 Department of Veterinary Medicine, College of Agriculture and Veterinary Medicine, Qassim University, P.O. Box 6622, Buraidah, 51452 Saudi Arabia; 2 Department of Biological Sciences, Kent State University, Kent, OH 44242 USA; 3 School of Biomedical Sciences, Kent State University, Kent, OH 44242 USA; 4 Department of Natural Sciences, Malone University, Canton, OH 44709 USA; 5 Lerner Research Institute, Cleveland Clinic, Cleveland, OH 44195 USA

Correction to: *Scientific Reports* 10.1038/srep37435, published online 25 November 2016

This Article contains an error where Tariq I. Almundarij is incorrectly affiliated with ‘College of Agriculture and Veterinary Medicine, Al-Qassim University, Buraydah, Al-Qassim Province, Saudi Arabia’. The correct affiliations for Tariq I. Almundarij are listed below:

Department of Veterinary Medicine, College of Agriculture and Veterinary Medicine, Qassim University, P.O. Box 6622, Buraidah 51452, Saudi Arabia

Department of Biological Sciences, Kent State University, Kent, OH, 44242, US

